# Complete Genome Sequence of the Extremely Thermoacidophilic Archaeon* Acidianus manzaensis* YN-25

**DOI:** 10.1128/genomeA.00438-17

**Published:** 2017-06-22

**Authors:** Ya-long Ma, Jin-lan Xia, Yun Yang, Zhen-yuan Nie, Hong-chang Liu, Li-zhu Liu

**Affiliations:** a School of Minerals Processing and Bioengineering, Central South University, Changsha, China; b Key Laboratory of Biometallurgy, Ministry of Education, Central South University, Changsha, China

## Abstract

The complete genome of *Acidianus manzaensis* YN-25 consists of a chromosome of 2,687,463 bp, with a G+C content of 30.62% and 2,746 coding DNA sequences. This archaeon contains a series of specific genes involved in the oxidation of elemental sulfur and reduced inorganic sulfur compounds.

## GENOME ANNOUNCEMENT

Bioleaching exploits acidophilic microorganisms to recover valuable metals, for instance, Au and Cu, from ores in the processes conducted at temperatures ranging from ambient to 80°C (1, 2). Higher-temperature operations with consortia of extremely thermoacidophilic archaea can improve the yield of target metals and reduce the problematic passivation on ore surfaces. *Acidianus manzaensis* is a novel thermoacidophilic archaeon of the *Acidianus* genus which is capable of oxidizing elemental sulfur and reduced inorganic sulfur compounds (RISCs) (3, 4). This strain has shown excellent capacity for metal recovery and high efficiency of chalcopyrite bioleaching (5–7). To investigate the mechanism that enables *A. manzaensis* to survive and proliferate during the biohydrometallurgical processes and to further research relevant metabolic pathways, such as sulfur oxidation, we performed whole-genome sequencing of *A. manzaensis* YN-25.

The strain *A. manzaensis* YN-25 was isolated from an acid thermal spring in Tengchong, China (4). The cells were cultivated with 9K basic medium [3.0 g/liter (NH_4_)_2_SO_4_, 0.5 g/liter MgSO_4_·7H_2_O, 0.5 g/liter K_2_HPO_4_, 0.1 g/liter KCl, 0.01 g/liter Ca(NO_3_)_2_] added with 0.2 g/liter yeast extract as a growth factor. Elemental sulfur was used as the electron donor (170 rpm at 65°C). The genomic DNA of* A. manzaensis* was extracted by the TIANamp DNA kit (Tiangen, Beijing, China) and sequenced using a combined strategy of the Illumina HiSeq 4000 (Illumina, USA) and PacBio RSII (Pacific Biosciences, USA) platforms (8). The genome sequence was assembled using the SOAP*denovo* software (9). The coding DNA sequences (CDSs) were predicted by Glimmer (10). Function annotation was completed by homologous comparison of each putative gene against the public databases, including the Kyoto Encyclopedia of Genes and Genomes, the Clusters of Orthologous Groups of proteins, Swiss-Prot, the nonredundant protein database, and Gene Ontology. In addition, rRNAs and tRNAs were predicted by RNAmmer and tRNAscan-SE, respectively.

The *A. manzaensis* YN-25 complete genome consists of a circular chromosome of 2,687,463 bp, with a G+C content of 30.62%. The genome is predicted to contain 2,746 CDSs. The number of tandem repeat sequences is 110 (6,113 bp), which makes up 0.2275% of the genome. The numbers of tRNAs and rRNAs are 42 and 2, respectively.

*A. manzaensis* YN-25 encodes several enzymes involved in the oxidation of sulfur and RISCs, including the sulfur oxygenase-reductase, thiosulfate:quinone oxidoreductase, and tetrathionate hydrolase. Electrons generated from sulfur and RISC oxidation enter the electron transport chain via quinone. Terminal quinol oxidases, like *aa*_*3*_ ubiquinol oxidase, receive electrons from quinone and transfer them to O_2_ coupled with ATP generation. Some electrons can be transmitted to the NADH complex to produce NADH by NADH-quinone oxidoreductase. The complete genome of *A. manzaensis* YN-25 provides novel insights into biologically catalyzed metal sulfide oxidation.

### Accession number(s).

The complete genome sequence of *A. manzaensis* YN-25 has been deposited in GenBank under the accession number CP020477. The version described in this paper is the first version, CP020477.1.

## References

[1] Rohwerder T, Gehrke T, Kinzler K, Sand W 2003 Bioleaching review part A: progress in bioleaching: fundamentals and mechanisms of bacterial metal sulfide oxidation. Appl Microbiol Biotechnol 63:239–248. doi:10.1007/s00253-003-1448-7. 14566432

[2] Vera M, Schippers A, Sand W 2013 Progress in bioleaching: fundamentals and mechanisms of bacterial metal sulfide oxidation—part A. Appl Microbiol Biotechnol 97:7529–7541. doi:10.1007/s00253-013-4954-2. 23720034

[3] Yoshida N, Nakasato M, Ohmura N, Ando A, Saiki H, Ishii M, Igarashi Y 2006 *Acidianus* *manzaensis* sp. nov., a novel thermoacidophilic archaeon growing autotrophically by the oxidation of H_2_ with the reduction of Fe^3+^. Curr Microbiol 53:406–411. doi:10.1007/s00284-006-0151-1. 17066338

[4] Ding JN, Zhang RY, Yu YZ, Jin DC, Liang CL, Yang Y, Zhu W, Xia JL 2011 A novel acidophilic, thermophilic iron and sulfur-oxidizing archaeon isolated from a hot spring of tengchong, Yunnan, China. Brazil J Microbiol 42:514–525. 10.1590/S1517-838220110002000016 PMC3769825 24031663

[5] He H, Xia JL, Yang Y, Jiang H, Xiao CQ, Zheng L, Ma CY, Zhao YD, Qiu GZ 2009 Sulfur speciation on the surface of chalcopyrite leached by *Acidianus* *manzaensis*. Hydrometallurgy 99:45–50. doi:10.1016/j.hydromet.2009.06.004.

[6] Zhu W, Xia JL, Yang Y, Nie ZY, Zheng L, Ma CY, Zhang RY, Peng AA, Tang L, Qiu GZ 2011 Sulfur oxidation activities of pure and mixed thermophiles and sulfur speciation in bioleaching of chalcopyrite. Bioresour Technol 102:3877–3882. doi:10.1016/j.biortech.2010.11.090. 21194927

[7] Liu HC, Xia JL, Nie ZY, Ma CY, Zheng L, Hong CH, Zhao YD, Wen W 2016 Bioleaching of chalcopyrite by *Acidianus* *manzaensis* under different constant pH. Miner Eng 98:80–89. doi:10.1016/j.mineng.2016.07.019.

[8] Jeong H, Lee DH, Ryu CM, Park SH 2016 Toward complete bacterial genome sequencing through the combined use of multiple next-generation sequencing platforms. J Microbiol Biotechnol 26:207–212. doi:10.4014/jmb.1507.07055. 26464377

[9] Li R, Zhu H, Ruan J, Qian W, Fang X, Shi Z, Li Y, Li S, Shan G, Kristiansen K, Li S, Yang H, Wang J, Wang J 2010 *De novo* assembly of human genomes with massively parallel short read sequencing. Genome Res 20:265–272. doi:10.1101/gr.097261.109. 20019144 PMC2813482

[10] Delcher AL, Bratke KA, Powers EC, Salzberg SL 2007 Identifying bacterial genes and endosymbiont DNA with glimmer. Bioinformatics 23:673–679. doi:10.1093/bioinformatics/btm009. 17237039 PMC2387122

